# Salivary Duct Carcinoma: Case Reports and Brief Review of the Literature

**DOI:** 10.1155/2021/2672772

**Published:** 2021-10-11

**Authors:** Deepti Kantamani, Sai S. Bandaru, Jennifer L. Miatech, M. Patrick Stagg

**Affiliations:** Baton Rouge General Medical Center-Bluebonnet, Internal Medicine Residency Program, Baton Rouge, LA, USA 70809-3679

## Abstract

Salivary duct carcinoma (SDC) is an uncommon and highly aggressive tumor associated with high morbidity and mortality. According to the World Health Organization, it is an extremely rare malignancy with an estimated incidence of 1-1.2 in 1,000,000 patients. Standard treatment for SDC is wide surgical resection along with lymph node dissection followed by adjuvant radiation therapy. The role of adjuvant chemotherapy is not known. In this report, we present three cases of SDC. A 71-year-old female with T1N0M0 disease was treated with total parotidectomy, ipsilateral neck dissection, and adjuvant radiotherapy without evidence of disease recurrence at 5 months. The second is a 59-year-old female with TXN1M0 disease who was treated with total parotidectomy with ipsilateral level I-IV neck dissection and adjuvant radiotherapy without evidence of disease occurrence at 21 months. The third case is a 79-year-old male with widely metastatic disease, including brain metastases, treated with cranial irradiation, leuprolide, and lapatinib who remains under home hospice care.

## 1. Introduction

Salivary duct carcinoma (SDC) is a rare, highly aggressive malignancy. The term SDC describes the malignancy that histologically resembles in situ and invasive ductal carcinoma of the breast. The parotid gland is the most commonly involved salivary gland accounting for approximately 80% of tumors, followed by the submandibular gland (8-12%) and minor salivary glands less than 10% [1, 2]. SDC is an extremely rare malignancy with an estimated incidence of 1-1.2 in 1,000,000 patients, with a higher prevalence in men [3]. It is often diagnosed at an advanced stage as it metastasizes early to regional lymph nodes and distant sites. Immunohistochemically, tumor cells are diffusely positive for cytokeratin 7 and androgen receptor stains and may be GATA binding protein 3 positive. HER2/neu positivity occurs in 15-40% of patients with SDC [2]. The mainstay of treatment for SDC is wide surgical resection along with lymph node dissection followed by adjuvant radiation therapy. The role of adjuvant chemotherapy and targeted therapies has limited benefit to date. Overall survival of the metastatic disease is poor, and 60-80% of patients with advanced-stage (T4) die within three years [4].

Adeberg et al. listed 1282 cases of SDC found in the literature between 1987 and 2019 [3]. Most clinical studies of SDC in the literature are institution-based retrospective cohort studies. This malignancy poses a challenge for physicians to provide education and information to the patients regarding the overall prognosis and treatment options given the absence of consensus guidelines. Due to the rarity of this devastating disease, we feel that each case should be reported. We describe three cases of SDC recently encountered in our institution.

## 2. Case Presentation

### 2.1. Case I

A 71-year-old African American female with no significant past medical history presented to her primary care physician in September 2020 for progressive swelling in her right parotid region for 3 months associated with a recent onset of dental pain. She denied any previous history of tobacco use or prior radiation exposure. Antibiotics resolved her dental pain without improvement of the right parotid swelling. She was then evaluated by head and neck surgery. Computed tomography (CT) of the head and neck revealed a 1.9 cm heterogeneously enhancing lesion in the anterior, superior superficial parotid gland without any enlarged nodes. She did not have any preoperative facial nerve involvement or lymphadenopathy. She underwent a total parotidectomy with clinically uninvolved margins. The complete facial nerve was dissected free of the neoplasm with gross preservation of function. Postoperatively, she had some weakness in the marginal mandibular branch of the facial nerve, along with weakness in closing her superior palpebral fissure of the right eye. Pathology of resected lesions revealed salivary duct carcinoma that was 1.7 cm in maximum dimension, positive for cytokeratin 7, HER2/neu equivocal 2+, and androgen receptor-positive. The tumor was negative for p40, mucin, estrogen receptor, progesterone receptor, TTF-1, SOX100, mammaglobin, p63, and s-100. HER2/neu was 1+ FISH not amplified. She had no lymphovascular invasion, but perineural invasion was present. Postoperative positron emission tomography (PET) scan before radiation was negative for metastatic disease. The tumor was staged pathologically as T1N0M0 stage I. She received adjuvant radiotherapy incorporating margin positivity with 6600 cGy in 30 fractions to the entire parotid bed and the adjacent nodes with 6000 cGy in 30 fractions. The remainder of her right neck received 5400 cGy in 30 fractions. She completed radiotherapy in January 2021. She developed facial dermatitis and dysgeusia as a result of radiation, but otherwise tolerated therapy. Currently, she is being followed with CT scans every 3 months and is without evidence of disease recurrence to date.

### 2.2. Case II

A 59-year-old Caucasian female with a past medical history of essential hypertension and a 35 pack-year history of tobacco smoking who first noted a neck mass in December 2018 presented to a head and neck surgeon in May 2019 complaining of a painless left-sided neck mass. She did not have any associated localized or constitutional symptoms. Fine-needle aspiration of the left neck mass in May 2019 returned positive for malignant cells that could not be further classified. PET/CT in June 2019 revealed a 0.9 × 1.0 × 1.2 cm solitary ovoid mass inferior to the left distal portion of the parotid gland. Imaging could not reliably determine if the mass inferior to the parotid gland originated from the parotid gland. The tumor stage was TXN1M0. A core biopsy of the left neck mass on June 20, 2019, showed neoplastic cells but could not be classified further. On July 8, 2019, she underwent an excisional biopsy of the mass by her head and neck surgeon. Intraoperatively, the mass was positioned between the cervical branch and the inferior branch of the marginal mandibular nerve and was firmly adherent to both the nerves. The adherence to the mass necessitated the sacrifice of these nerves. The parotid had no abnormalities on palpation and no clinically positive margins. Several enlarged cervical lymph nodes found on resection were negative for malignancy. Final pathology revealed salivary duct carcinoma involving a single intraparotid or periparotid lymph node with a tumor measuring approximately 9 mm in greatest dimension and is present <1 mm from nearest tissue edge. The tumor cells were positive for GATA-3, BRST-2, cytokeratin 7, and HER2/neu and showed focal scattered positivity for S-100, p63, and p40. Immunohistochemical stains were positive for mammaglobin and androgen receptors. There was no extranodal extension identified. On July 17, 2019, the patient underwent left total parotidectomy and ipsilateral level I-IV neck dissection with excision of sublingual gland and a submandibular gland biopsy. The final pathology showed benign salivary gland tissue in the left parotid, left submandibular gland, and left sublingual gland. All lymph nodes removed from left level I-IV were negative for malignancy. Postoperatively, she developed paresthesia of the left lateral tongue and left lower lip. She completed adjuvant radiotherapy to the postoperative parotid bed and ipsilateral neck with a total dose of 60/57/54 cGy in 30 fractions on October 7, 2019. She developed xerostomia and hypothyroidism as a result of radiation therapy. The patient declined adjuvant platinum-based chemotherapy and targeted therapy. She is followed every 3 months with serial imaging without evidence of disease to date.

### 2.3. Case III

A 79-year-old Caucasian male presented to his primary care physician for routine follow-up in November 2020. He has a past medical history of insulin-dependent diabetes mellitus, essential hypertension, hyperlipidemia, and coronary artery disease with coronary artery bypass grafting and pacemaker placement. He denied any previous history of tobacco use. On physical examination, a nontender mass on the left side of his neck was easily palpable. Complete review of systems was otherwise negative except for increased forgetfulness. CT scan of the neck revealed a large mass arising from or immediately adjacent to the left parotid gland. Multiple left lung masses representing metastatic disease were also present on this scan. PET scan revealed 2.9 × 3.4 cm hypermetabolic soft tissue mass in the left parotid gland and extensive metastatic disease in the lung, mediastinum, liver, and bones. He underwent an ultrasound-guided biopsy of the left parotid mass and a CT-guided biopsy of a left lung nodule. Pathology revealed salivary duct carcinoma in the parotid and metastatic disease in the lung. Immunohistochemistry was positive for cytokeratin 7, GATA-3, and mammaglobin. HER2/neu and androgen receptors were positive in the lung biopsy. Magnetic resonance imaging (MRI) of the brain contained numerous ring-enhancing lesions, approximately 40-50. He received whole-brain radiation 30 cGy in 10 fractions in December 2020. He tolerated radiation therapy without complication. Following a discussion about goals of care, the patient decided against palliative chemotherapy. He did agree to androgen deprivation therapy with leuprolide. Next-generation sequencing identified no actionable targets except for HER2/neu amplification. He started lapatinib in February 2021. Despite dual therapy with leuprolide and lapatinib, he developed new visual symptoms prompting a repeat MRI of the brain on May 20, 2021, which revealed a mixed response to therapy. The majority of the central nervous system enhancing metastatic lesions decreased in size or resolved compared to the previous MRI on December 8, 2020. However, several new lesions were slightly more prominent than the previous ones. The patient decided to transition to comfort care and has been under home hospice since May 2020.

## 3. Discussion

Kleinsasser et al. described salivary duct carcinoma (SDC) in 1968 as a salivary malignancy that histologically resembles in situ and invasive ductal carcinoma of the breast [2, 5]. The World Health Organization recognized it as a distinct tumor type in 1991. SDC is rare, accounting for approximately 1 to 3% of all malignant salivary gland tumors [2]. Most tumors arise de novo, although 20% of cases arise secondarily from preexisting benign pleomorphic adenoma (i.e., carcinoma ex pleomorphic adenoma) [6]. The most common presenting symptom is a painless neck mass. Locally advanced disease can involve the facial nerve resulting in facial weakness. According to the case series published by Jaehne et al., which included 50 cases, around 66% present at T3/T4 stage [7, 8]. SDC predominantly affects male patients (66%-75%), presenting with a wide age range from 34 to 83 years and a mean age of diagnosis at 62.5 [8].

SDC is a highly aggressive high-grade salivary malignancy with a tendency for early regional, distant metastasis, and a high-grade recurrence. The most common sites of distant metastasis are the lung, bone, brain, and liver. Perineural invasion, lymphovascular invasion, extracapsular spread, higher N stage, and facial nerve involvement often are considered negative prognostic indicators, although findings are inconsistent. Cheng et al. described that perineural invasion and LVI were frequently present in approximately 57-69% and 61-70% of patients, respectively. Extranodal invasion occurred in 58% of patients [2]. The series published by Jaehne et al. found local disease recurrence in 48% of patients at 17.4 months after initial treatment and distant metastases in 48% after an average of 28 months [8]. The mean survival of patients in this report was 36 to 56 months. The 5-year survival rate for stage I disease was 42%, stage II was 40%, stage III was 30.8%, and stage IV was 23.2% [8]. Patients with parotid gland involvement had a better prognosis than those with submandibular or minor salivary gland involvement.

Currently, no National Comprehensive Cancer Network (NCCN) guidelines for the specific treatment of SDC exist. NCCN guidelines recommend complete surgical excision of tumor for major salivary gland tumors without nodal involvement (N0) with or without neck dissection for high-grade and T3/T4 salivary gland tumors. The recommendation for those with node involvement (N+) is complete surgical resection combined with neck dissection. In addition, they recommend adjuvant radiotherapy for high-risk features like intermediate or high grade, close or positive margins, neural/perineural invasion, lymph node metastasis, lymphatic/vascular invasion, and T3-4 tumors. Postoperative radiotherapy for SDC is an appropriate therapeutic option regardless of stage and margin status [9]. Excellent local control rates can be achieved with extensive (local) surgical treatment and postoperative RT [10]. Smaller trials discuss the activity of chemotherapy regimens including a taxane plus platinum. Randomized clinical trials demonstrating the benefit of cytotoxic chemotherapy are lacking.

SDC treatment is most often with targeted therapy. Androgen receptor expression is seen in over 90% of the cases of SDC, making the tumor a potential target for androgen deprivation therapy [9]. Additionally, HER2 expression occurs in between 15 and 40% of cases [2]. Therapy with trastuzumab for HER2 overexpressing tumors improves overall survival [9]. A case series of 50 tumor samples published by Jaehne et al. showed that the intensity of HER2/neu expression correlates with the early development of distant metastasis and poor 5-year survival rate. The 2-year, 3-year, and 5-year survival in patients with HER2 1+ were 88.9%, 55.6%, and 11.1% compared to 51.4%, 17.1%, and 0%, respectively, in those with HER2 3+ positivity [8]. Further research regarding androgen deprivation therapy and other targeted therapy is warranted. There are no consensus guidelines in the management of metastatic disease.

We report three cases of SDC. Case 1 is clinically stage 1, T1N0M0 treated with total parotidectomy with ipsilateral neck dissection and adjuvant radiotherapy. She is under close monitoring postradiotherapy and has remained disease-free since January 2021 to date. Case 2 is clinically stage 2, TXN1M0 treated with total parotidectomy with ipsilateral level I-IV neck dissection and adjuvant radiotherapy who remains disease-free since October 2019. Case 3 presented with widely metastatic disease and remains under home hospice care after undergoing cranial irradiation and treatment with leuprolide and lapatinib.

## 4. Conclusion

The rarity of salivary duct carcinoma and lack of consensus guidelines necessitates the reporting of these cases. We present three cases including staging, therapy, and outcome data to enhance our understanding of this disease and build a more substantial data set for historical controls in future clinical trials. Finally, we hope clinical trials employing robust historical data will soon address the treatment of metastatic SDC, which remains an unmet medical necessity.
